# Automation Use and Dis-Use in Golf: The Impact of Distance Measuring Devices on Trust in Technology and Confidence in Determining Distance

**DOI:** 10.3389/fpsyg.2021.655387

**Published:** 2021-07-02

**Authors:** Lori Dithurbide, Heather F. Neyedli, Jamie Swinimer, Jamie MacFarlane

**Affiliations:** School of Health and Human Performance, Dalhousie University, Halifax, NS, Canada

**Keywords:** trust, technology, performance, reliance, golf, information automation

## Abstract

An athlete’s decision to use technology depends on trust in the automation, and confidence in their abilities. Distance measuring devices (DMD) are used in golf to estimate yardage. The purpose of these studies was to examine how DMD usage affects trust in the DMD, confidence in determining yardage manually, and golf performance over time. In study 1, DMD non-users played four rounds of golf, two with the DMD and two without. In study 2, DMD users played five rounds, three with the device, and two without. Participants’ trust in automation, confidence, and performance were recorded by online survey at baseline and following each round. Giving a DMD to non-users influenced trust in automation and confidence. When DMD users relinquished the device, confidence decreased briefly but rebounded quickly, trust in automation was unaffected. Performance was unchanged in both groups. These studies provide information about how confidence in abilities and trust in automation interact.

## Introduction

In the modern world of sport, the use of technology has become a prevalent and growing theme. Many sports such as football, baseball, basketball, and golf use technology or automation to aid performance, assist in training, and assist officials in decision-making. In golf, a common piece of technology used to aid athletes is a range finder or GPS system. These systems are also referred to as distance measuring devices (DMDs). The use of these devices is currently allowed in all amateur championship competitions (The R&A, 2020) unless stipulated by a tournament organizer, however, they are generally not currently permitted in professional play.

A DMD is a form of information automation, where the system has functions of sensing and registering data (Parasuraman et al., 2000). A DMD provides a golfer with information relating to distance in order to aid in their club selection and their shot execution. Automation can aid performance and/or reduce workload; however, this is only the case if the human operator (i.e., golfer) relies on it appropriately. Reliance on automation may be dependent on factors such as how much the operator trusts the automation and how much the operator trusts their own capabilities (Lee and See, 2004; Hoff and Bashir, 2015). Although there is an abundance of research on trust in automation in other domains (e.g., aviation, process control), to date, there is little research in the context of sport. Technology use in sport will continue to grow, therefore it is important to better understand how humans interact with technology, and why they may choose, or not, to use it. DMDs are a popular tool for golfers around the world and it is important to understand the impact of their use.

Trust in automation is defined as “the attitude that an agent will help achieve an individual’s goals in a situation characterized by uncertainty and vulnerability” (Lee and See, 2004, p. 54). Based on the model of trust in technology from Lee and See (2004), the potential user may actively or passively gather information about the technology, which forms their initial basis for trust. Trust then influences the potential user’s intention of whether to use the technology. Use of the technology provides feedback, which then can update the user’s trust in the system. Conversely, if the decision has been made to not use technology, the user still may update their beliefs about the system based on their own performance without use of the technology.

Another factor that may be influenced by the automated system is operator’s self-confidence. Self-confidence is a term used to describe a person’s belief in their ability to perform a desired behavior or accomplish a certain level of performance. Self-confidence is a term that envelopes models and theoretical concepts such as self-efficacy, perceived confidence or ability, sport-confidence, and movement confidence (Feltz, 2007). Self-confidence has frequently been shown in research to have a large impact on an athlete’s performance (Feltz, 2007). In fact, athletes with higher levels of self-confidence tend to be more effective at using cognitive resources for successful performance in sport (Hays et al., 2009). This means that golfers who are more confident in their abilities to estimate yardage may be able to more effectively evaluate factors that influence club selection and shot execution, the same factors a DMD can influence. Furthermore, individuals with greater confidence in their own abilities may not form the intention to use technology because they feel like they may achieve equal or better performance without investing in the technology.

Another factor relating to self-confidence is the loss of manual skills, which is a common concern with the introduction of automation into other domains. For instance, in aviation, the proliferation of automation in the cockpit has led to a reduction in the manual skills needed to fly and navigate the aircraft (Ebbatson et al., 2010), and cognitive, information-based skills may show a greater decrement compared to motor skills (Casner et al., 2014). Reduced manual skills following the removal of automation is indicative of over reliance on the automation. Within the context of golf, there is a potential for golfers to lose the manual skill of estimating yardage, which may impact their overall performance. Another possibility is that the precise distance information available from the DMD may lead the user to perceive their performance at estimating yardage as inferior to the technology, reducing their confidence in estimating yardage.

Within the context of the use of DMD devices in golf, both confidence and trust were affected by whether a golfer owned a DMD or not. In a recent study by Dithurbide and Neyedli (2019), the researchers conducted a survey that examined factors that influence a golfer’s decision to use a DMD or not. Golfers who used a DMD had lower confidence in their own abilities and greater trust in the DMD compared to non-users. This research was the first to indicate that there may be a relationship between the use of technology in sport and the user’s trust in automation and their self-confidence; however, due to the cross-sectional design, the directional nature of the relationship was unclear. More specifically, it cannot be determined whether DMD users purchased the device because they were more predisposed to trust technology or whether the use of the DMD increased their trust in the device through feedback about the device’s performance. Similarly, it cannot be determined whether DMD users purchased the device because they had low confidence in their own abilities or whether use of the device lowered their confidence in their own abilities. Further, there is a gap in knowledge on how the introduction of the DMD in non-users, or the removal of the DMD in users, impacts trust in the technology, self-confidence in one’s abilities, and golf performance. While it is not feasible to follow golfers in their voluntary choices to choose to purchase or use DMDs (or choose not to), the most viable next step in this line of research is to examine the implications of experimentally manipulating the use or disuse of the device and examining the impact on trust in the technology, confidence in one’s own abilities, and performance.

Consequently, the purpose of this research was to examine how DMD usage affects trust in the DMD, confidence in one’s own abilities to determine yardage, and golf performance over time. Specifically, we aimed to determine how the introduction or removal of a DMD to golfers affects their trust in DMDs, their confidence in estimating yardage, and their golf performance. Study 1 examined how the introduction of a DMD to golfers who do not use DMDs affects their trust in DMDs, their confidence in their own abilities to estimate yardage, and their golf performance. It was hypothesized that the use of DMDs in non-users would have an effect on the golfers’ trust in DMDs, their confidence in their own abilities to estimate yardage, and their golf performance. If the introduction of a DMD did affect trust and confidence, based on Dithurbide and Neyedli (2019) findings, we expected to see trust increase and confidence decrease. No directional hypothesis was proposed for golf performance. Study 2 examined how the removal of a DMD to golfers who consistently use a DMD affects their trust in the DMD, their confidence in their own abilities to estimate yardage, and their golf performance. It was hypothesized that the removal of a DMD would have an effect on trust in the DMD, trust in their own abilities to estimate yardage and golf performance (non-directional alternative hypotheses). Results from this research will inform further research on how athletes interact with technology in sport performance and training, and the impact of this interaction on athlete psychological factors. Two studies were conducted for an efficient examination of two aspects of the technology-user relationship: adding technology to a non-user, and removing technology from a habitual user. Both are reflective of real-world scenarios: Study 1 reflects a golfer’s adoption of a DMD, such as if the golfer was given a DMD as a gift. Study 2 reflects no longer being able to use the DMD, which is particularly relevant to golfers approaching higher levels of competition, where DMDs are not prohibited, and the DMD user would need to adapt to not relying on their device but also can apply to golfers of all levels if they forget their device or it runs out of charge.

## Materials and Methods

### Study 1—DMD Non-users

#### Participants

An *a priori* power analysis using the smallest observed effect size associated with DMD use from Dithurbide and Neyedli (2019) determined that a minimum of 10 participants was needed to achieve a power level of 0.8 to detect the effect of DMD use. A total of 18 (17 males and 1 female) golfers were recruited for the study and 15 complete data sets were obtained (i.e., questionnaires completed after all rounds) and included in the analysis. Participants were on average 56.11 years of age (range = 25–78 years of age), with an average handicap factor of 12.31 (range = 4.4–19). Each participant lived in or near the Halifax Regional Municipality and was a registered member of Golf Canada. These criteria were set to ensure that the researchers could meet with participants in person prior to their participation and to ensure that participants had a valid handicap and allowed their golf scores to be tracked. A golf handicap factor or index is a numerical measure of a golfer’s potential, where better golfers have lower handicaps (i.e., a handicap factor of 5 indicates greater golfing ability than a handicap of 10). It should be noted that this research was conducted prior to the World Handicap System being launched in January 2020. In order to participate, golfers needed to have a valid handicap of 20 or lower. This criterium was set given that previous research (Dithurbide and Neyedli, 2019) indicated that golfers with a handicap above 20 typically did not believe they were skilled enough for the use of a DMD. On average, participants had 32.05 years of golfing experience, while 50% of the participants indicated they played 20–40 rounds each year while the remaining 50% indicated they played more than 40 rounds each year. Lastly, the participants did not own or typically use a DMD, thus making them eligible to participate in the study.

The participants were recruited using communications through a provincial golf association, and from individual clubs. If an individual was interested in participating, they were asked to contact the first author directly via email. The first author then distributed the informed consent form to the potential participant and screened them for the aforementioned inclusion and exclusion criteria. This study received Research Ethics Board approval prior to any recruitment efforts, from the Social Science and Humanities Ethics Board at Dalhousie University, file 2017–4207.

#### Study Design and Procedure

##### Study Design

This study was a repeated measures, pre-test and post-test design, during which the participants completed baseline measurements, collected during an initial meeting and over two rounds of golf. The baseline measurements were then followed by subsequent measurements over two additional rounds of golf, and a 1-month follow-up following the completion of all four rounds of golf. All four rounds of golf took place within 1 month. The participants completed the study at any golf course of their choice.

##### Procedure

Once the participants were recruited and agreed to participate in the study, an initial meeting took place at a location of their choice. During this initial meeting, the informed consent was collected according to the Research Ethics Board of the authors’ institution and approval, and the participant completed the demographics and experience, confidence in abilities, and trust in automation measures. Participants were also provided with a DMD and instructed on the basic uses of the device. This device was to be used during their third and fourth rounds of golf within the study, and participants were asked to make adopting the DMD the only substantial change they made to their game during their participation in the study.

Following the initial meeting, the participants played two rounds of golf without a DMD, as they typically would. For these two rounds participants were instructed to not alter anything around their typical golf performance. After each round and using the online survey platform Opinio, the participants reported their score and completed the confidence in abilities measure. Participants were asked to fill out this online survey as soon as they could upon completing their round. Participants were then instructed to use the provided DMD for their third and fourth rounds of golf. Following each of the next two rounds of golf (third and fourth rounds), the participants reported their golf score and completed both the confidence in abilities and trust in automation measures. As with the previous rounds, participants were asked to complete these online questionnaires as soon as they could following their round of golf. One-month following the completion of all four rounds, the participants were then asked to complete the 1-month follow-up questionnaire, also using Opinio.

#### Materials and Measures

##### Distance Measuring Device

The DMD that was used by the participants in this study was the NEO GHOST by Bushnell Golf. This simple pocket-sized device uses GPS technology and provides information to the user regarding the distance to the front, middle, and back of the green.

##### Demographics and Experience

Participants were asked to provide demographic information including their age, gender, and location of home course. Participants were also asked to provide information including their handicap factor, how frequently they play golf, and how many years they have been playing. The participants also provided information, as an open-ended response, on the factors that have influenced their decision to not use a DMD.

##### Trust in Automation

A modified validated scale to measure trust in automation (Jian et al., 2000) was used to examine the participants’ trust in DMDs. For the purpose of this study and similar to Dithurbide and Neyedli (2019) the wording of Jian et al. (2000) scale on trust in automation was altered to fit the context of golf. For example, on the original scale one of the items reads, “the system is deceptive.” This item was modified to read, “the range finder/GPS does not always provide me with good information to benefit my game decisions.” Another item on the scale was altered from “the system’s actions will have a harmful or injurious outcome” to “the range finder/GPS’s information will decrease my performance.” In previous research (Wang et al., 2009; Neyedli et al., 2011) similar modifications have been made to specifically fit the purpose of the research. From the large sample from Dithurbide and Neyedli (2019), the scale had high internal constancy (Cronbach’s α = 0.91). This questionnaire contained 12 items and each item was measured on a 7-point Likert scale, in which a score of 1 corresponded to complete disagreement and a score of 7 corresponded to agreement with a specific item on the questionnaire.

##### Confidence

Participants were asked to complete a measure assessing their confidence in their ability to estimate yardage without the use of automation. This questionnaire was modified from the trust in automation questionnaire while maintaining consistent questions, where possible, to ensure that the wording was relevant to a golfer’s confidence in their own manual ability to determine yardage. For example, the third item in the trust in automation questionnaire was “I am suspicious of the range finder/GPS’s outputs.” This item corresponds to “I am not confident in my own estimates of yardage” in the confidence in abilities questionnaire. The second item [“The range finder/GPS works in a concealed manner (i.e., I do not understand the process by which the range finder works”)] and eleventh item (“I am familiar with the range finder/GPS”) on the confidence in automation questionnaire were not modified and were excluded as items in the confidence in abilities questionnaire due to the lack of ability to modify them to relate to confidence in estimating yardage. The same questionnaire was used in Dithurbide and Neyedli (2019) and also had high internal constancy (Cronbach’s α = 0.94). This questionnaire contained 10 items and each item was measured on a 7-point Likert scale, in which a score of 1 corresponded to complete disagreement and a score of 7 corresponded to agreement with a specific item on the questionnaire.

##### Golf Scores

Golf scores were used as a measure of golf performance. Each participant reported their score following each round of golf that they played. Golf scores are recorded in reference to par (i.e., +10 for a score of 82 on a par-72 course) to account for courses with different pars.

##### One-Month Follow-Up

In the 1-month follow-up questionnaire, the participants were asked if they have continued to use a DMD, and if not, what the likelihood was that they would purchase or start to use one in the near future. This likelihood was measured on a 7-point Likert scale with a score of 1 corresponding to “very unlikely” and a score of 7 corresponding to “very likely.” The participants were also asked to complete an open-ended question on what the factors were that have influenced their decision to use (or plan to use) a DMD.

#### Data Analysis

For both the trust and confidence questionnaires, the reverse scored questions were inverted. The average score across all questions was then calculated for each round for each participant. Performance was measured by score to par.

All measures were analyzed using separate one-way repeated measures ANOVAs with round as the independent variable. There were different numbers of levels (i.e., Rounds) for each measure. For the confidence value, the value at baseline and for the following four rounds were entered into the ANOVA. For trust, the value at baseline and for Round 3 and 4 (the two rounds in which the participants used the device and trust was measured) were entered into the ANOVA. For score, the four rounds were entered into the ANOVA. To follow up on a significant effects simple contrasts were used to compare the value on each round to the baseline score.

### Study 2—DMD Users

#### Participants

Participants were recruited using similar methods as Study 1; however, local media unexpectedly picked up the story of the study during recruitment for Study 2, through a reporter receiving the recruitment email from the provincial golf association resulting in a greater number of participants. A total of 34 (21 males and 13 females) golfers were recruited for the study with 28 complete data sets included in the analysis. Participants were on average 62.47 years of age (range = 35–72 years of age), with an average handicap factor of 11.39 (range = 4.9–20). Each participant lived in or near the Halifax Regional Municipality and was a registered member of Golf Canada. The same criteria were set for participation in this study as was in Study 1, except for Study 2, participants must have indicated that they consistently use (i.e., on most holes and for every round) a DMD. On average, participants had 35.24 years of golfing experience, while only 2 of the participants indicated they played 20–40 rounds each year while the remaining 32 participants indicated they played more than 40 rounds each year. Participants indicated that they had been using a DMD for a mean time of 6.91 years. The first author distributed the informed consent form to the potential participant and screened them for the aforementioned inclusion and exclusion criteria. This study also received Research Ethics Board approval prior to any recruitment efforts, from the Social Science and Humanities Ethics Board at Dalhousie University, file 2018–4526.

#### Study Design and Procedure

##### Study Design

This study was a repeated measures, pre-test and post-test design, during which the participants completed baseline measurements, collected during an initial meeting and over two rounds of golf. The baseline measurements were then followed by subsequent measurements over three additional rounds of golf, and a 1-month follow-up following the completion of all five rounds of golf. All five rounds of golf took place within 1 month. The participants completed the study at any golf course of their choice.

##### Procedure

Once the participants were recruited and agreed to participate in the study, an initial meeting took place at a location of their choice. During this initial meeting, the informed consent was collected according to the Research Ethics Board of the authors’ institution and approval, and the participant completed the demographics and experience, confidence in abilities, and trust in automation measures.

Following the initial meeting, the participants played two rounds of golf with their own DMD, as they typically would. Participants were asked to play these rounds without making any alterations to their game. After each round and using the online survey platform Opinio, the participants reported their score and completed the confidence in abilities and trust in automation measures. Participants were instructed to complete these questionnaires as soon after the round as they were able to. Participants were then instructed to stop using their DMD for their third and fourth rounds of golf. Participants were asked to make the removal of the DMD the only substantial change they made to their game at this time. Following these next two rounds of golf (third and fourth rounds) without the use of the DMD, the participants reported their golf score and completed both the confidence in abilities and trust in automation measures. As with the first two rounds, participants were asked to fill out the questionnaires as soon after the round as possible. Participants were then instructed to play a fifth and final round of golf, this time using their DMD once again as they usually would. Participants reported their golf score and completed both the confidence in abilities and trust in automation measures once again following this fifth round. One-month following the completion of all five rounds, the participants were then asked to complete the 1-month follow-up questionnaire using Opinio.

#### Materials and Measures

##### Distance Measuring Device

Participants were instructed to use their own DMDs. DMDs can vary in type and model, and in technology (e.g., GPS and laser), however, all provide information regarding the distance from the golfer and their target. Participants were instructed to use their own DMDs so as not to alter their trust in the DMD, and confidence both in their own abilities and the DMD itself during the participation in the study. Participants reported having used their DMDs for an average of 6.91 years. The most common responses to why they used DMDs included improving accuracy, improving club selection, and having received the DMD as a gift.

##### Questionnaires

Demographic questions, confidence in abilities, trust in automation, and golf score were all measured in the same way as Study 1. In the 1-month follow-up questionnaire, the participants were asked if they have continued to use their DMD (yes or no), how much they missed using their DMD in rounds three and four (Likert scale 1 = do not miss at all to 7 = missed it very much), and how likely they would be to purchase another DMD should they lose their current one (Likert scale 1 = very unlikely to 7 = very likely). The participants were also asked to provide additional comments in an open-ended question box the two above Likert questions.

#### Data Analysis

Trust, confidence and score to par were calculated the same way as Study 1. Again, each of the measures were entered into separate one-way repeated measures ANVOAs with round as the independent variable. For confidence and trust, the baseline value and the trust values for all five rounds were entered into the ANOVA. For score to par, the score for all five rounds was entered into the ANOVA. Similar to Study 1, to follow up on a significant effects simple contrasts were used to compare the value on each round to the baseline score.

## Results

### DMD Non-users

**FIGURE 1:**
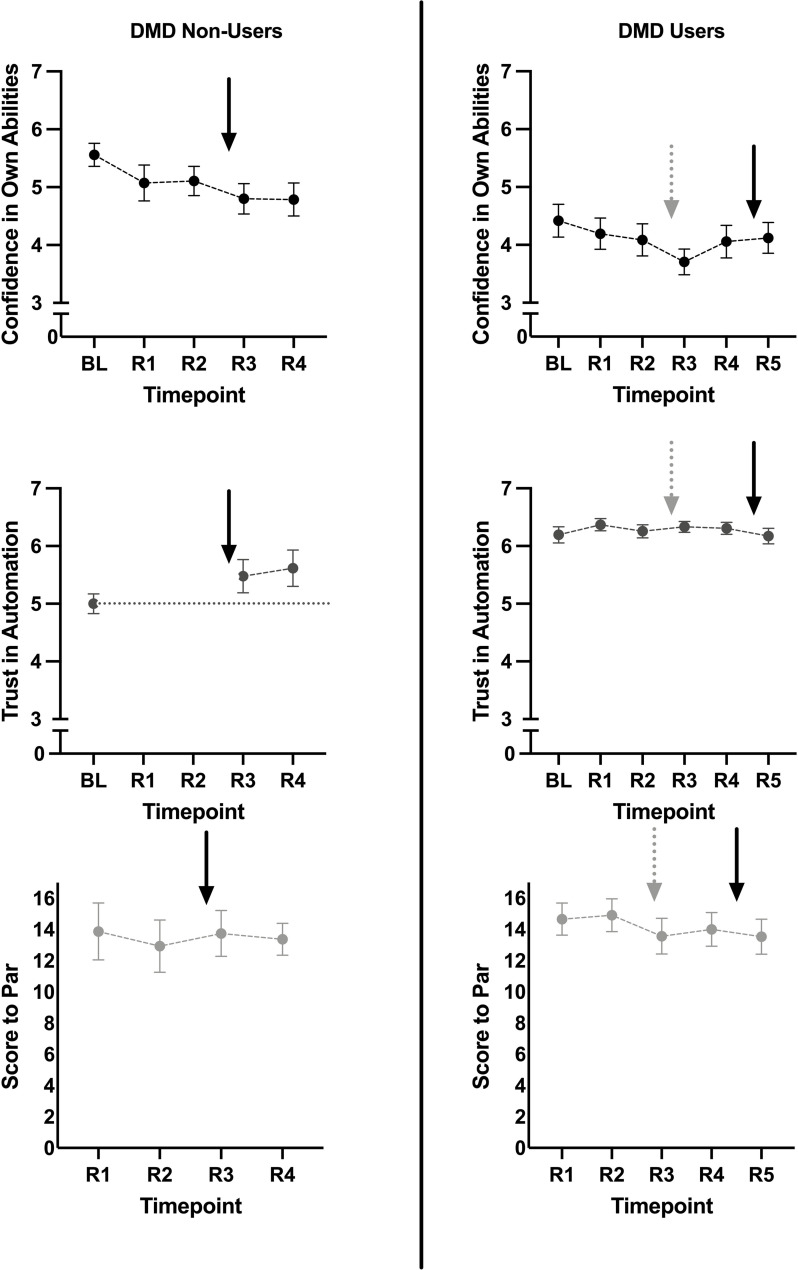
Plots show change over time in confidence in abilities, trust in automation, as well as golf score to par (top to bottom, respectively). Black solid arrows indicate the re-introduction of a DMD. Gray dotted arrows indicate the removal of a DMD. Error bars are SEM.

On average across rounds, the internal consistency of the confidence measure was good (average Cronbach’s α = 0.89). There was a significant effect of Round on the participant’s confidence in their own ability to estimate yardage, *F*(4, 56) = 2.75, *p* = 0.037, partial-η^2^ = 0.16 (Figure 1). Compared to Baseline, participants demonstrated lower confidence after the two rounds in which they used the DMD, [Round 3, *F*(1, 14) = 7.94, *p* = 0.014, partial-η^2^ = 0.36; Round 4, *F*(1, 14) = 8.72, *p* = 0.01, partial-η^2^ = 0.38]. The other two rounds were not significantly different than Baseline [Round 1, *F*(1, 14) = 2.91, *p* = 0.11, partial-η^2^ = 0.17; Round 2, *F*(1, 14) = 2.65, *p* = 0.13, partial-η^2^ = 0.16].

On average across rounds, the internal consistency of the trust measure was good (average Cronbach’s α = 0.87). There was an effect of Round on the participant’s trust in the DMD, *F*(2, 28) = 4.05, *p* = 0.029, partial-η^2^ = 0.22 (Figure 1). Trust in the DMD was significantly higher than Baseline in Round 4, *F*(1, 14) = 5.45, *p* = 0.035, partial-η^2^ = 0.20. While trust was higher than baseline in Round 3, the difference did not reach significance, *F*(1, 14) = 3.51, *p* = 0.082, partial-η^2^ = 0.20. There was no significant effect of Round on Score to Par, *F*(3, 45) = 0.304, *p* = 0.80, partial-η^2^ = 0.02 (Figure 1).

**FIGURE 2:**
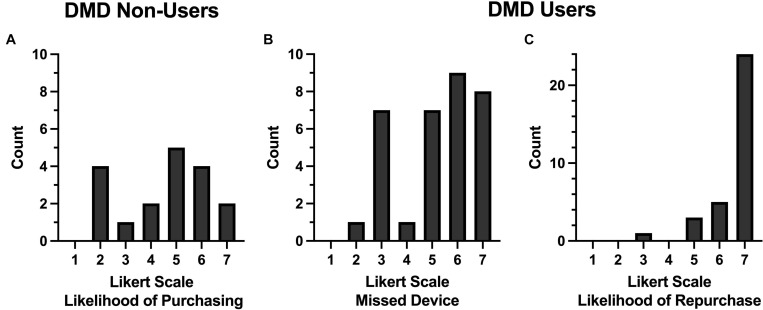
Histograms of the number of participants who provided a particular response on a seven-item Likert scale to follow-up questions. Note the different *y*-axis scale used in C. **(A)** DMD non-users indicated on a scale of 1 (very unlikely) to 7 (likely) how likely they would be to purchase a DMD. **(B)** DMD users indicated how much they missed their DMD when asked to play without it on a scale of 1 (did not miss at all) to 7 (miss it very much). **(C)** DMD users indicated on a scale of 1 (very unlikely) to 7 (very likely) how likely they would purchase another DMD.

Responses and descriptive statistics from the 1-month follow-up indicated that only one participant began using a DMD consistently following the participation in the study. Further, for those participants who had not yet began to use a DMD, when asked to indicate on a scale of 1 (very unlikely) to 7 (likely) how likely they would be to start using a DMD or purchasing one in the near future, the sample was fairly split with a mean of 4.41 and range between 2 and 7 (Figure 2A). Participants also varied in their responses to an open-ended question asking participants what factors influence their decision to purchase a DMD in the future or not. For those participants who do not intend to purchase or use in the near future, factors such as confidence in their own ability, experience at their home course, no perceived impact on their performance, being comfortable in their own routines, or cost of the device were mentioned. For those participants who were more likely to purchase or use a device in the near future, factors such as added confidence in their decision making (i.e., club selection), greater accuracy in yardage estimation, added value when playing courses they are not familiar with, and to speed up pace of play.

### DMD Users

Again, on average across rounds, the internal consistency of the confidence measure was excellent (average Cronbach’s α = 0.95). There was a significant effect of Round on participant’s confidence in their own ability to estimate yardage, *F*(3.16, 85.5) = 2.72, *p* = 0.047, partial-η^2^ = 0.09 (Figure 1). Round 3, where the participants stopped using the DMD, had lower confidence compared to Baseline, *F*(1, 27) = 7.05, *p* = 0.013, partial-η^2^ = 0.21. None of the other Rounds were significantly different than Baseline, [Round 1, *F*(1, 27) = 2.58, *p* = 0.12, partial-η^2^ = 0.09; Round 2, *F*(1, 27) = 3.86, *p* = 0.06, partial-η^2^ = 0.125; Round 4, *F*(1, 27) = 2.00, *p* = 0.17, partial-η^2^ = 0.07; Round 5, *F*(1, 27) = 1.85, *p* = 0.19, partial-η^2^ = 0.06].

Again, on average across rounds, the internal consistency of the confidence measure was good (average Cronbach’s α = 0.84). There was no significant effect of Round on trust in the DMD, *F*(3.77, 98.1) = 0.872, *p* = 0.48, partial-η^2^ = 0.03 (Figure 1). There was also no main effect of Round on score to par, *F*(4, 124) = 0.95, *p* = 0.44, partial-η^2^ = 0.03.

Participants were asked in the follow-up survey to indicate how much they missed their DMD when asked to play without it (Rounds 3 and 4 of the protocol). Participants responded on a scale of 1 (did not miss at all) to 7 (miss it very much). Results showed that many participants did miss their DMDs with a mean response of 5.21, with a range of responses from 2 to 7, and a mode response of 6 (Figure 2B). When asked to provide an optional open-ended comment for this item, participants mentioned that they missed using the DMD the most when they were closer to the targets (i.e., green) where accuracy is most important, or if they were hitting from a position where distance to the target was more difficult to determine (e.g., off the fairway and not near yardage markers in the middle of the fairway). Participants also commented that they missed the comfort and confidence the accuracy provided them. However, some participants commented on how they did not miss it as much as they thought they would and that not having a DMD did not impact their score as much as they had anticipated.

All participants continued using a DMD following their study participation at the 1-month follow up. When asked to indicate on a scale of 1 (very unlikely) to 7 (very likely) how likely they would purchase another DMD in the event they would lose or break their current device, participants overwhelmingly indicated that they would very likely do so (Figure 2C). When asked to provide an optional open-ended comment for this item, participants mentioned that the DMD was especially helpful when playing unfamiliar courses, that they rely heavily on the information it provides and that the information provides comfort and confidence, and that the use of a DMD speeds up pace of play.

## Discussion

The purpose of these studies was to examine the influence of the experimental manipulation of DMD usage on trust in the DMD, confidence in one’s own abilities to determine yardage, and golf performance over time. Specifically, we aimed to determine how the introduction or removal of a DMD to golfers affects their trust in DMDs, their confidence in estimating yardage, and their golf performance. Study 1 examined the influence of a DMD on the aforementioned variables when introduced to golfers who did not use a DMD, while Study 2 examined the influence of removing a DMD in golfers who regularly used one. Study 1 found that introducing the DMD to non-users affected both their trust in automation and their confidence in their own abilities, though there was no effect on golf performance. Study 2 demonstrated that when regular DMD users stopped using their device, their confidence in their ability to determine yardage on their own decreased for one round. However, there were no changes in golf performance or trust in automation, despite the removal of the DMD in Rounds 3 and 4, and the resumption of DMD use in Round 5.

These findings support the hypothesis that the use of a DMD increases trust in the DMD. Trust is an important factor in human reliance on automation. Through a dynamic interaction, reliance on automation influences trust and trust influences reliance on automation (Lee and See, 2004). This means that if a particular device is not trusted then it is not likely to be used, and if it is not used then it is not likely to be trusted. In prior work, Dithurbide and Neyedli (2019) found that golfers who own DMDs have greater trust in automation than golfers who do not own DMDs; however, their study did not distinguish whether the use of (or reliance on) DMDs is influencing golfers’ trust in DMDs, or golfers’ trust in DMDs is influencing the use of DMDs. In the current study, introducing DMDs to non-users increased trust in device. This is in line with previous research that shows that some form of reliance on automation is a key first step in order for trust to grow because it allows a user to observe and learn about the behavior of the automation (Muir and Moray, 1996). Conversely, the removal of a DMD did not significantly impact the trust in automation of users. Given that the participants in Study 2 (DMD users) had an average of 6.91 years of experience using a DMD, this could indicate some resiliency within trust in automation beliefs. It is possible that observing their own performance and confidence when not using the device reinforced their level of trust in the automation, which was high in this group. Another possibility is that due to the length of experience users had with their DMD, two rounds of golf without were not enough to alter those trust beliefs.

In terms of confidence in one’s own abilities, the current studies demonstrate that the presence of a DMD (or lack thereof) has some influence on a golfer’s confidence in their ability to estimate yardage. The DMD user group saw an immediate decline in confidence when their DMD was removed, however, this rebounded quite quickly. This could indicate an immediate feeling of vulnerability when the assistance of automation is removed. Without the DMD, the golfer now has to rely on their own ability, perhaps after not having done so in a substantial amount of time, causing doubt in their abilities. It is interesting that confidence in abilities increased in Round 4, possibly indicating the adjustment period to a golfer employing their own distance estimates without the use of a DMD. The use of a DMD corresponded with a decline in confidence in the non-user group, which does fit with existing literature. Those who use DMDs were shown to have lower confidence in their own abilities (Dithurbide and Neyedli, 2019), which are in line with the findings here, that the introduction of a DMD would lead to a decrease in one’s ability specific confidence. The literature does not address whether the golfers owned DMDs because they had less confidence, or they had less confidence because they owned DMDs. This current study brings to light evidence that the latter may be true and that golfers may have less confidence in their own abilities to estimate yardage as a result of reliance on DMDs.

The purpose of a DMD is to help golfers estimate the appropriate distance to a particular target. In the current studies, actual golf performance did not significantly vary based on the presence or lack thereof of a DMD. DMD use affected participant’s confidence and confidence is consistently shown in the literature to influence one’s performance (Feltz, 2007). In golf specifically, this is poignant given the mental resources needed to perform, and the association between higher levels of confidence and more effective use of one’s cognitive capabilities (Hays et al., 2009). Despite this, the results of this study suggest an acute change in DMD use has minimal impact on one’s overall golf performance. In golf, to date, there is little to no research on the effects that automation, specifically DMDs, have on performance in the sport. Automation has been shown to improve human performance factors such as efficiency and safety, and reduce operator workload in other contexts such as health-care, transportation, and at home (Wickens et al., 2015). These findings may not be entirely applicable here as the golfers are entirely capable of judging the distance themselves, as evidenced by the DMD user group. The adjustment of confidence also seems to indicate that the reliance on the DMD is not based on workload reduction or optimization, but a workload easement, which does not truly influence the performance of a task. Further and in support of the suggestion that DMDs lead to workload easement, many participants reported in the follow-up questionnaire that they wanted to continue to use device because it increased the pace of play (i.e., they played faster). Golfers may have felt that the pace of play was increased due to the reduction of time and/or effort spent estimating yardage without the DMD. In addition to this, the DMD only measures yardage, and as such, the golfer still must account for factors such as the course layout and weather manually, meaning the golfer still must be highly involved in the task of club selection and shot execution.

Another possibility is that there is an effect on performance, but the influence is small enough variability introduced by other factors (e.g., weather conditions, etc.) masked the effect. Furthermore, the impact of the DMD device may emerge over time. Measuring performance over a longer period of time following the introduction or removal of the device or measuring yardage estimation performance directly, may yield different results by both reducing the noise associated with other factors that influence overall performance results and by increasing the amount of time spent with or without the device.

One limitation of this study is that the subjects only participated for 1 month and across four and five rounds of golf. This may indicate that the increase in trust in DMDs and decreased confidence in ability to estimate yardage may only be an acute, short-term result. The fact that golf performance (i.e., golf score to par) did not significantly change throughout the study may also be because of the short duration of participation. Future research should look at a longer participation time and across more rounds of golf to see if the results trend in the same direction. The possibility of the change in confidence being an acute effect based on a sudden change in the use of automation is supported by the fact that confidence did increase between non-DMD rounds in the user group.

Another limitation of this study is that there was only one female identifying participant in Study 1. This means that the results of that study cannot be generalized to both men and women. Dithurbide and Neyedli (2019) found that women had higher trust in technology then men. With more women as participants, perhaps the results of this study would demonstrate a greater increase in the trust in automation measure. As golf is played by individuals of all genders, it is also important that the research conducted reflects the population of individuals playing the sport. Future research should attempt to collect a more generalizable sample with respect to gender, or at least collect a sample of mostly women to compare with the findings of this study. Other individual factors may affect trust in automation and confidence; for example, experience, skill, and age, which were not included in the current study analysis. Future research should examine how these individual factors may impact automation usage in sport.

Another individual factor that should be considered in future work is how a user acquired or began using a DMD. Study 1 was intended to mimic the “gift scenario,” where an individual is gifted a DMD and begins to use it regularly. This phenomenon is supported by findings from Study 2, where many participants stated their reasoning for beginning to use a DMD is that they received the device as a gift. However, what was not considered in this body of research is the possibility of differences in trust and confidence in the technology between a user who is given the technology to use, compared to an individual who actively decides to seek out said technology. Assessing differences in trust and confidence in technology across users who sought the device versus being gifted the equipment would make for an interesting future addition to this area of study; however, this limitation does not seriously impede the results seen in the present studies, as participation was voluntary and specifically mentioned using a DMD, so participants likely had at least some desire to try using the DMDs. Furthermore, neither study captures the characteristics of golfers who have tried a DMD and then decided to stop. Future research could examine what influences a person’s decision of whether to continue using the device including factors such as trust and characteristics of the device itself. Given that devices seem to have similar and reasonable accuracy, it is likely that different features of the device or cost have an influence on whether a person chooses to use and continues to use a particular device. That being said, unbiased and scientific validation studies on the accuracy of different DMDs will be important in better understanding why golfers choose to begin or continue using a DMD.

The current study did not include a control group. This means that the results cannot indicate any causal relationships between the introduction of DMDs to non-users and the variables assessed in this study. A true control group is difficult to implement given that there is no placebo device that can be given to participants. Giving a device that provided sham (i.e., false information) may actually lead to decreases in trust. This was the rationale for including two rounds prior to the introduction or removal of the device to demonstrate the stability of these measures, particularly confidence prior to the introduction or removal of the device.

The use of technology in sport by athletes, coaches and sport organizations is likely to continue to increase. Technological advances occur at a rapid pace. Athletes, coaches, and sport organizations continually strive to improve and push the boundaries of human performance. Consequently, advancing knowledge on how humans interact with technology and the impact of these interactions is integral in the practical application and usage of technology in training and competition.

The results of this study would suggest implications to an athlete’s level of confidence when technology is first introduced, as well as when technology is initially removed after consistent use. Because the context of this study is in golf, specifically in determining distance to a target, golfers should be cautious to not over rely on their DMDs. This is especially true for those golfers aspiring to progress to professional golf, where DMD usage is not yet permitted. Maintaining the ability and confidence in determining yardage on their own may be helpful in transitioning to competitive play without the use of a DMD. Results from the open-ended follow-up questions in Study 2 suggest that participants adjusted to playing without a DMD fairly quickly and this is supported by the statistical analysis from Study 2.

Follow-up questions also indicated that cost of a device is an important factor in whether or not to purchase a DMD. DMDs can vary in cost (approximately $50–500 CAD) and capabilities. Many non-users stated that the potential benefit of the DMD did not outweigh its cost, while some users indicated the opposite. This could also potentially be impacted or moderated by the golfer’s confidence in their own abilities to estimate yardage. Consequently, a golfer’s financial resources may also impact whether or not they use a DMD.

The results of this study imply that continued work in acquiring and building confidence in an athlete’s own abilities in information gathering and decision-making during training or competition is integral to an athlete’s perception of performance. While technology may provide more information and at a greater specificity and accuracy, how that information is used and applied is also important. In sports such as golf, where technology provides information and the user must still execute a skill (i.e., information automation), athletes must still have the ability and confidence in the information they are provided and the information they gather themselves. Therefore, in order to best support these athletes, better understanding the interactions and impacts of technology on the athlete may lead to more accurate reliability on technology, on themselves, and consequently, better performance.

## Conclusion

This study is one of the first studies to look at human interaction with automation in the context of sport and golf. It adds to previous research conducted by Dithurbide and Neyedli (2019) and is the first study to look at the effects of introducing DMDs to non-users or removing the DMD from users. This study provides information on the dynamic relationship between trust in automation and confidence in one’s own abilities. Furthermore, this study provides a bridge in the trust in automation literature in a sport context between cross sectional designs, such as prior work done by Dithurbide and Neyedli (2019), and more longitudinal work, necessary to understand the causation of these relationships. Future research can use this study as a foundation when looking further into how automation affects golfers trust in automation, confidence, and performance. This study is positioned in the context of golf, but the results seen here may have value outside of a sport context as well. Trust in automation, confidence in one’s abilities, and how these concepts relate to an individual’s performance are relevant to understand in a plethora of other areas, such as in healthcare and the automotive industry.

## Data Availability Statement

The raw data supporting the conclusions of this article will be made available by the authors, without undue reservation.

## Ethics Statement

The studies involving human participants were reviewed and approved by the Dalhousie University Research Ethics Board. The patients/participants provided their written informed consent to participate in this study.

## Author Contributions

LD was responsible for the conceptualization of the research, contributing to the research design, responsible for participant recruitment and data collection, and primary contributor to the writing of the manuscript. HN contributed to the conceptualization of the research and its design and is the primary contributor to the data analysis, also contributed to the writing of the manuscript. JS contributed to the data management and analysis of Study 2 and contributed to the writing and formatting of the manuscript. JM contributed to the data management and analysis of Study 1 and contributed to the writing of the manuscript. All authors contributed to the article and approved the submitted version.

## Conflict of Interest

The authors declare that the research was conducted in the absence of any commercial or financial relationships that could be construed as a potential conflict of interest.
